# Special Issue: Sulfonamides

**DOI:** 10.3390/molecules22101642

**Published:** 2017-09-29

**Authors:** Claudiu T. Supuran

**Affiliations:** Dipartimento Neurofarba, Sezione di Scienze Farmaceutiche e Nutraceutiche, Università degli Studi di Firenze, Via Ugo Schiff 6, I-50019 Sesto Fiorentino (Florence), Italy; claudiu.supuran@unifi.it

The sulfonamides and their structurally related derivatives, such as the sulfamates and sulfamides, possess the general formula A-SO_2_NHR, in which the functional group is either directly bound to an aromatic, heterocyclic, aliphatic, or sugar scaffold (of type A), or appended to such a scaffold via a heteroatom, most frequently oxygen or nitrogen (leading thus to sulfamates and sulfamides, respectively) [1,2,3,4]. The nature of the R moiety may also be quite variable, starting with hydrogen, case in which primary sulfonamides/sulfamates/sulfamides are being considered [5], and ranging to a variety of moieties incorporating heteroatoms (OH, NH_2_, etc.) as well as organic scaffolds of the types mentioned above for A [6,7]. As thus, this class of compounds may lead to a huge range of derivatives, which are generally easily available through classical synthetic methodologies [5,6,7], and in addition, possess drug-like properties, well-known for decades [8,9,10,11,12,13,14,15].

Indeed, the sulfonamides constitute an important class of drugs, with many types of pharmacological agents possessing antibacterial [4], anti-carbonic anhydrase [2,8,9,10,11,12], anti-obesity [13], diuretic [14,15], hypoglycemic [16], antithyroid [17], antitumor [18,19,20], and anti-neuropathic pain [21] activities, among others. The common chemical motifs present in the aromatic/heterocyclic/sugar/amino acid sulfonamides endowed with such properties is thus associated with a multitude of biological activities, and many others are being constantly reported, such as, among others: matrix metalloproteinase and bacterial protease inhibitors [22,23], HIV protease inhibitors [24], non nucleoside HIV reverse transcriptase or HIV integrase inhibitors [25,26], etc. This is probably due to the particular features of the -SO_2_NH- (or -OSO_2_NH-, -NHSO_2_NH-) moieties, which can participate in multiple interactions with metal ions, amino acid residues, DNA or RNA moieties present in various biomolecules acting as drug targets [27,28,29,30]. Furthermore, sulfonamides and their isosteres are generally stable, easy to prepare and bioavailable, which may explain the huge number of drugs incorporating these motifs [7,8,9,10,11,12,13,14,15,16,17,18,19,20,21,22,23,24,25,26].

The following special issue of *Molecules* is in fact a nice example of this multitude of possible applications of the sulfonamides, with the wide range of targets to which they bind, diverse synthetic procedures and pharmacological applications, some of which highly innovative, for many representatives of this interesting class of pharmacologic agents. The first contribution is a nice review article [31] from Silvestri’s group, dealing with N-pyrrylarylsulfones, a class of pharmacological agents discovered using the sulfonamides as leads, through a simplification of the functional group. The extensive review presents both the many synthetic procedures for obtaining representatives of this class, as well as many relevant examples of their biological activity as antiviral, anticancer and SNC drugs [31].

Considering the fact that the sulfonamides were the first antibacterials [4,32], due to their interfering with dihydropteroate synthase and dihydrofolate reductase enzymes from bacteria (and protozoa) [32,33] the next two papers from the special issue deal with this type of applications of sulfonamides incorporating sulfa drugs in their molecules, such as sulfadiazine [34] or sulfamethoxazole [35]. The first paper describes hybrids incorporating sulfonamides (such as sulfadiazine) to which other chemotypes have been attached, e.g., ciprofloxacin (an antibacterial agent [36]) or amantadine (an antiviral [3]). These hybrids were tested as inhibitors of jack bean urease, some of them showing low nanomolar activity. Both kinetic and computational studies were performed in order to investigate the inhibition mechanisms of these new sulfonamides [34]. The paper by Krátký et al. [35] describes another interesting hybrid drug approach in the search of new anti-mycobacterial agents. Thus, sulfamethoxazole has been derivatized at its primary amino moiety by using alkyl isocyanates, with the formation of a large series of ureas. Other derivatives were synthesized by reacting sulfamethoxazole with oxalyl chloride. These sulfonamides were tested as inhibitors of the growth of several *Mycobacterium* species, such as *M. avium*, *M. kansassii*, some of them showing remarkable activity [35].

The next three papers in the special issue [37,38,39] deal with targeting carbonic anhydrases (CAs) from various organisms [1,2,8,9,10,11,12]. Indeed, these metalloenzymes are potently inhibited by various classes of sulfonamides, many of which show pharmacologic applications as antiglaucoma [8,10], antiobesity [13], antitumor [8,9,11,18], or diuretic [15] drugs. The first contribution by Vullo et al. [37] presents an interesting work on the cloning and purification of β- and γ-class CAs from the pathogenic bacterium *Burkholderia pseudomallei*, and the inhibition of these enzymes with a range of more than 40 sulfonamides and sulfamates. Indeed, due to the relevant problem of drug resistance to commonly used antibiotics, the inhibition of CAs from pathogenic organisms started to be considered as an alternative, innovative approach for finding new such pharmacologic agents [40,41].

The next paper [38] presents an optimization for the synthesis of sulfonamide CA inhibitors derived from 1,3,5-triazine, aromatic sulfonamides and amino acid derivatives. This class of CA inhibitors was reported earlier to represent highly efficient and isoform-selective compounds for the tumor-associated CA isoforms IX and XII over the cytosolic, widespread CA I and II [42,43,44]. In the present paper, the authors present and alternative synthesis in which the base used earlier (a tertiary amine) [42,43] was replaced by sodium carbonate in aqueous medium, leading to a better yield in the desired sulfonamide [38].

In the paper by Berrino et al. [39] a new series of benzenesulfamide derivatives (-NH-SO_2_NH_2_) which incorporate a 1-benzhydrylpiperazine tail, connected to the sulfonamide scaffolf by means of β-alanyl or nipecotyl spacers was reported and investigated for the inhibition of CAs of human (h) origin, such as hCA I, II, IV and IX. Some of these isoforms are established drug targets, but many sulfonamide or sulfamide inhibitors show little selectivity when inhibiting them. Some of the new sulfamides reported in this paper did show some selective inhibitory profile, mainly against hCA I, which has been rationalized by using computational methods [39].

The next paper in the special issue [45] investigates another enzyme, lactoperoxidase, for its interaction with sulfonamides incorporating (poly)acetoxybenzamide and/or (poly)hydroxybenzamide scaffolds. These secondary sulfonamides were effective-medium potency lactoperoxidase inhibitors, with inhibition constants varying between the nano- to the micromolar range [45].

Marciniec et al. [46] present on the other hand a highly interesting paper in which acetylenic quinolone sulfonamides are prepared by an innovative synthetic approach, followed by testing of their antiproliferative activity against several breast cancer cell lines. Many of these derivatives showed potent antitumor activity, comparable to that of cisplatin, and are thought to bind to some cytochrome P450 isoforms, for two of which computational studies were presented [46].

In the paper by Lin et al. [47] sulfadiazine is again used as the main scaffold, to which gallic acid moieties were introduced in order to obtain agents with pro-chondrogenic effects for the treatment of cartilage diseases. Gallic acid was thus derivatized with the sulfonamide moiety present in the sulfa drug sulfadiazine in order to increase the hydrophobicity and the bioavailability of this agent. Although the mechanism of action of this agent is not clearly understood so far, it seems that it interferes with the activity of a disintegrin and metalloproteinase with thrombospondin motifs 5 (ADAMTS-5) [47].

In conclusion, the present special issue presents an interesting collection of high quality papers which underline the many potential applications of the simple, sulfonamide structural motif, a highly used, almost magic moiety in the tool kit of medicinal chemists.
